# A retrospective cohort study on red blood cell morphology changes in pre-school age children under nitrous oxide anesthesia

**DOI:** 10.1186/s12871-021-01388-5

**Published:** 2021-06-16

**Authors:** Ruoxi Wang, Ling Lan, Li Xu, Bo Zhu, Yuguang Huang

**Affiliations:** 1 Department of Anesthesiology, Peking Union Medical College Hospital, Chinese Academy of Medical Sciences and Peking Union Medical College, Beijing, China; 2 Department of Anesthesiology, Peking Union Medical College Hospital, No.1 Shuaifuyuan, Dongcheng District, Beijing, 100730 China

**Keywords:** Nitrous oxide, Child, preschool, Red blood cells

## Abstract

**Background:**

Megaloblastic anemia or bone marrow changes could occur after prolonged nitrous oxide inhalation via vitamin B_12_ inactivation related DNA synthesis impairment. Previous researches have studied hematological changes with nitrous oxide exposure, but only in adults or adolescents. Pre-school age children with active hematopoietic red bone marrow are more vulnerable to potential side effects of nitrous oxide and might experience growth impairment. The purpose of our study was to analyze red blood cell morphology changes under nitrous oxide anesthesia in pre-school age children.

**Methods:**

One hundred thirty-six children under 5 years old scheduled for hemivertebra resection were analyzed. According to fresh gas type in anesthesia records, 71 children who received nitrous oxide in oxygen during anesthesia maintenance were categorized into the nitrous oxide group and the other 65 who received air in oxygen were the air group. Complete blood counts in perioperative period were assessed for anemia, macrocytosis, microcytosis, anisocytosis, hyperchromatosis and hypochromatosis. The peak value and change percentage were calculated for mean corpuscular volume and red cell distribution width.

**Results:**

Forty-two children in the air group (64.6%) and 30 in the nitrous oxide group (42.3%) developed anemia (*P* = 0.009). None developed macrocytosis in both groups. Postoperative mean corpuscular volume peaked (mean [95% confidence interval]) at 83.7(82.9–84.4) fL, and 83.2(82.4–83.9) fL and postoperative red cell distribution width at 13.8% (13.4–14.2%), and 13.9% (13.6–14.2%) for the air group and the nitrous oxide group. Both the relative change of mean corpuscular volume (*P* = 0.810) and red cell distribution width (*P* = 0.456) were similar between the two groups.

**Conclusions:**

No megaloblastic red blood cell changes were observed with nitrous oxide exposure for 4 h in pre-school age children undergoing hemivertebra resection.

## Background

Nitrous oxide (N_2_O), an inexpensive and odorless gas with low solubility in blood, is widely used for induction and maintenance of anesthesia especially in children and outpatients [1]. Recent studies have shown increased plasma homocysteine concentration in adults [2], adolescents [3] and children [4] under N_2_O-based anesthesia, as the result of methionine synthase inhibition via vitamin B_12_ oxidation. Some significant hematologic complications like megaloblastic bone marrow changes have been reported in critically ill patients or those with vitamin B_12_, folate or methionine synthetic disorders under N_2_O exposure [5]. While in adolescents under major spinal surgery, N_2_O exposure was not associated with megaloblastic anemia [6].

Bone cavities in pre-school age children are filled with active hematopoietic red bone marrow that is vulnerable to vitamin B_12_ and other nutrients deficiency. With increased respiratory rate and shorter drug elimination half-life time, young children are prone to receive larger doses of inhaled anesthetics. Since the side effects of N_2_O are dose-dependent, children undergoing prolonged major surgery are more likely to develop macrocytosis and megaloblastic anemia, which might be accompanied by slower recovery and a longer hospital stay. Scoliosis with hemivertebra is one of the common congenital malformations in young children, and hemivertebra resection, a mature surgery by a specific team of professional surgeons in our hospital [7], is usually performed between 2 to 5 years old. Considering young children might be more vulnerable to N_2_O exposure, a retrospective cohort study on patients under 5 years old scheduled for hemivertebra resection was applied to analyze changes in red blood cell morphology under N_2_O anesthesia.

## Methods

### Design and setting

We retrospectively analyzed changes in red blood cell morphology in pre-school age children under hemivertebra resection. The retrospective cohort study was conducted in Peking Union Medical College Hospital, China, between January 2013 and January 2020, in accordance with the Declaration of Helsinki. Before data collection, the study was approved by the Medical Ethics Committee of Peking Union Medical College Hospital and the registration number was S-K1301. A waiver of informed consent was approved by the institutional review board of Medical Ethics Committee of Peking Union Medical College Hospital because it was a retrospective analysis. We wrote this manuscript according to The Strengthening the Reporting of Observational Studies in Epidemiology (STROBE) checklist.

### Study population

Children under 5 years old, scheduled for hemivertebra resection, with a postoperative hospital stay of more than 3 days and no history of liver or kidney disease were included in this study. This study excluded patients with a history of N_2_O exposure. And for those who underwent more than one such operation, only the first surgery was included. Patients with preoperative hematopoietic disease, and those without complete blood counts (CBCs) before surgery were also excluded.

### Anesthesia management

All patients received standard total intravenous general anesthesia as the clinical routine in our hospital. Some of them received short inhalation anesthesia to open venous access. Propofol (3 mg/kg), fentanyl (2 μg/kg) and rocuronium (0.6 mg/kg) were used for induction. General anesthesia was maintained with continuous infusion of propofol and remifentanil. Fentanyl boluses (1–2 μg/kg) were given when needed for intraoperative analgesia. The tidal volume was set at 8–10 mL/kg and the respiration rate was set around 20 times per minute with a fresh gas flow of N_2_O/O_2_ or Air/O_2_ 2 L/min at the discretion of the anesthesiologist. Some anesthetists prefer N_2_O-based anesthesia for the reduction of other sedative and analgesic drugs. Some were afraid of postoperative nausea and vomiting with N_2_O anesthesia and chose air instead. Intraoperative transcranial motor evoked potential was used during the surgery. Bispectral index score was maintained between 40 and 60, and the hemodynamic variables were controlled within 20% of baseline values. Lactated Ringer’s solution was used for fluid maintenance and blood products were administered when necessary. Intraoperative blood salvage and cell saver were used according to the estimation of bleeding by surgeons. Sufentanil or morphine was used via patient-controlled intravenous analgesia pump for postoperative analgesia. All patients received posterior hemivertebra resection and vertebral fusion in prone position by the same group of surgeons. A drainage tube was placed underneath paravertebral muscles before closing. After the operation, with adequate spontaneous ventilation and airway reflexes, all patients were extubated and back to postanesthesia care unit or ward.

### Measurements

Demographic, surgical and anesthesia data such as age, sex, height, weight, anesthesia time and operation time were collected through electronic medical record system and there were no missing data. CBCs before and up to 7 days after surgery were also retrieved. The baseline CBC was taken as the latest report before surgery. If there were more than one CBC report within 24 h, the average was calculated and recorded for that day. Since most of the reference intervals of parameters in CBC were similar between children aging 2 to 5 years old and adults [8], and the patients included were within this age range, the diagnostic criteria used in our hospital was thought to be appropriate in our study. Anemia was defined as hemoglobin < 110 g/L. Macrocytosis and microcytosis were defined as mean corpuscular volume (MCV) < 82 fL or > 97 fL. Anisocytosis was defined as red cell distribution width (RDW) > 15%. Hypochromatosis and hyperchromatosis were defined as mean corpuscular hemoglobin concentration (MCHC) < 320 g/L or > 360 g/L. The concentration and maintenance time of N_2_O were recorded. The cumulative exposure dose was calculated as the result of multiplying the concentration by the exposure time of N_2_O. The differences between the highest index during 7 days after surgery and the index before surgery were taken as the peak change of MCV and RDW (△MCV, △RDW). And the differences divided by preoperative values were recorded as the relative changes of △MCV and △RDW (r△MCV, r△RDW). Moreover, data were collected on intraoperative blood loss, fluid infusion and perioperative red blood cell transfusion. Different types of blood transfusion concluding autologous or allogeneic or mixed red cell transfusion were recorded and analyzed separately.

### Statistical analysis

The sample size of this study was limited by the time the electronic medical record system was used. Patients were divided into two groups according to the record of N_2_O use: patients who received N_2_O for the maintenance of anesthesia (N_2_O group), and patients who received air instead (Air group).

Demographic characteristics and perioperative data were described and analyzed firstly. The Kolmogorov-Smirnov test was applied to check for distribution normality. If the data was non-normally distributed, a two-tailed Mann-Whitney *U* test was used. And if the data was normally distributed a two-tailed unpaired *t* test was used. The amount of intraoperative blood loss, fluid infusion and red cell transfusion were analyzed by the Mann-Whitney *U* test. The fraction of different kinds of transfusion was calculated and Chi-squared test was used to determine significant differences between the two groups. The incidence of anemia, macrocytosis, microcytosis, anisocytosis, hypo- and hyperchromatosis were described and analyzed in the same way. The two-tailed unpaired *t* test was used to compare △MCV and r△MCV and the two-tailed Mann-Whitney *U* test was used to compare △RDW and r△RDW. To explore the association between cumulative N_2_O exposure and r∆MCV and r∆RDW, Spearman correlation coefficient and 95% confidence interval (CI) were calculated. Baseline factors thought to be associated with r∆MCV were regarded as potential confounders for analysis. Based on previous studies and clinical experiences, age, sex, blood loss amount [9], intraoperative fluid infusion, allogeneic transfusion amount and exclusively allogeneic transfusion percentage [10, 11] were adjusted in multiple liner regression analysis. IBM SPSS version 22 was used for statistical analysis, and a 2-tailed *P* value of < 0.05 was considered significant.

## Results

**Fig. 1:**
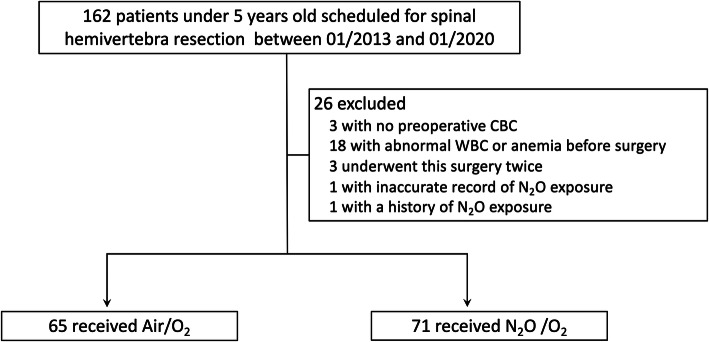
Flowchart. Legend: Flow chart showing inclusion and exclusion of patients for spinal hemivertebra resection. CBC, complete blood count; WBC, white blood cell; N_2_O, nitrous oxide

One hundred sixty-two patients were screened and in total 136 patients were included in this retrospective cohort study, of which 65 were in the air group and 71 were in the N_2_O group. The flow chart was shown in Fig. 1. The demographic characteristics before surgery were shown in Table 1. There was no significant difference between the two groups, except for the expected larger exposure dose in the N_2_O group.

**Table 1:** Demographic characteristics and perioperative data

	Air group	N_**2**_O group	***P*** value
Number of patients	65	71	
Male, N (%)	42 (64.6)	46 (64.8)	0.983
Age (months)	43.6 ± 14.4	43.5 ± 13.0	0.827
Weight (kg)	15.7 ± 4.1	16.1 ± 3.7	0.381
Height (cm)	97.3 ± 10.7	97.6 ± 10.7	0.789
Duration of surgery (min)	167.5 ± 37.1	172.4 ± 35.5	0.265
Duration of anesthesia (min)	233.6 ± 38.8	230.9 ± 40.0	0.912
N_2_O inspiration concentration (%)	n.a.	50 [50–50]	< 0.001
Cumulative N_2_O dose (N_2_O × time)	n.a.	100.5 [84.0–112.5]	< 0.001
Length of stay (days)	13.8 ± 4.2	13.6 ± 4.8	0.766
Preoperative red blood cell (10^12^/L)	4.6 ± 0.4	4.6 ± 0.3	0.828
Preoperative hemoglobin (g/L)	127.7 ± 9.1	126.7 ± 7.1	0.496
Preoperative white blood cell (10^9^/L)	7.0 ± 1.5	7.0 ± 1.4	0.945
Preoperative platelet (10^9^/L)	314.3 ± 73.3	293.9 ± 68.7	0.094

Data are presented as numbers (percentage), or mean ± standard deviation. *N*_*2*_*O* nitrous oxide; *n.a.* not applicable due to low count

Blood loss, fluid infusion and red cell transfusion during and after surgery were listed in Table 2. There was no difference in intraoperative blood loss and fluid infusion between the two groups. 127(93.4%) patients were transfused during surgery, among which 60(92.3%) were in the air group and 67(94.4%) were in the N_2_O group. 20(30.8%) and 34(47.9%) patients in air group and N_2_O group received only allogeneic blood transfusion (*P* = 0.042), and the transfusion volume was 200[200–400] and 200[200–400] respectively (*P* = 0.761). 7(10.8%) and 6(8.5%) patients in air group and N_2_O group received only autologous blood transfusion (*P* = 0.646) and the transfusion volume was 130[128–139] and 123[109–174] (*P* = 0.445). 33(50.8%) and 27(38.0%) patients in air group and N_2_O group received both kinds of transfusion (*P* = 0.135), and the transfusion volume was 349[327–516] and 350[325–514] (*P* = 0.761). Besides, only 2(3.1%) patients in the air group and 1(1.4%) patient in N_2_O group received blood transfusion after operation. Through the transfusion type were different between the two groups, there was no significant discrepancy in blood transfusion volume.

**Table 2:** Perioperative Blood Loss, Fluid Infusion and Blood Transfusion

	Air group	N_**2**_O group	***P*** value
Intraoperative blood loss (mL)	200 [125–300]	200 [100–300]	0.722
Intraoperative fluid infusion (mL)	500 [400–800]	540 [400–750]	0.948
No red cell transfusion, N (%)	5 (7.7)	4 (5.6)	0.737
Intraoperative red cell transfusion			
N (%)	60 (92.3)	67 (94.4)	0.737
Volume (mL)	325 [200–400]	270 [200–400]	0.780
Exclusively allogeneic transfusion			
N (%)	20 (30.8)	34 (47.9)	0.042
Volume (mL)	200 [200–400]	200 [200–400]	0.761
Exclusively autologous transfusion			
N (%)	7 (10.8)	6 (8.5)	0.646
Volume (mL)	130 [128–139]	123 [109–174]	0.445
Mixed transfusion			
N (%)	33 (50.8)	27 (38.0)	0.135
Volume (mL)	349 [327–516]	350 [325–516]	0.761
Postoperative red cell transfusion			
N (%)	2 (3.1)	1 (1.4)	0.606

Postoperative red cell transfusions were exclusively allogeneic. Data are presented as numbers (percentage), or median (25th–75th percentile). *N*_*2*_*O* nitrous oxide

The fraction of hematologic disorders was shown in Table 3. 42(64.6%) and 30(42.3%) patients in the air and N_2_O group developed anemia(*P* = 0.009). No macrocytosis happened, but 37(56.9%) and 37(52.1%) patients had microcytosis in the air group and the N_2_O group (*P* = 0.574). There was 1(1.5%) patient in the air group with hypochromatosis. 4(6.2%) and 3(4.2%) patients developed hyperchromatosis separately in the air group and the N_2_O group (*P* = 0.709). Besides 6(9.2%) and 5(7.0%) patients in the two groups developed anisocytosis. These results suggested a change in red blood cell morphology, mainly in hemoglobin and MCV. The blood loss and transfusion volume may be a contributing factor.

**Table 3:** Incidence of Abnormality in Red Blood Cell up to Seven Days after Surgery

	Air group	N_**2**_O group	***P*** value
Anemia (Hb < 110 g/L), *N* (%)	42 (64.6)	30 (42.3)	0.009
Macrocytosis (MCV > 97 fL), *N* (%)	0	0	–
Microcytosis (MCV < 82 fL), *N* (%)	37 (56.9)	37 (52.1)	0.574
Hypochromatosis (MCHC< 320 g/L), *N* (%)	1 (1.5)	0 (0)	0.478
Hyperchromatosis (MCHC> 360 g/L), *N* (%)	4 (6.2)	3 (4.2)	0.709
Anisocytosis (RDW > 15%), *N* (%)	6 (9.2)	5 (7.0)	0.640

Data are presented as numbers (percentage). *N*_*2*_*O* nitrous oxide; *Hb* hemoglobin; *MCV* mean corpuscular volume; *MCHC* mean corpuscular hemoglobin concentration; *RDW* red-cell distribution width

**Fig. 2:**
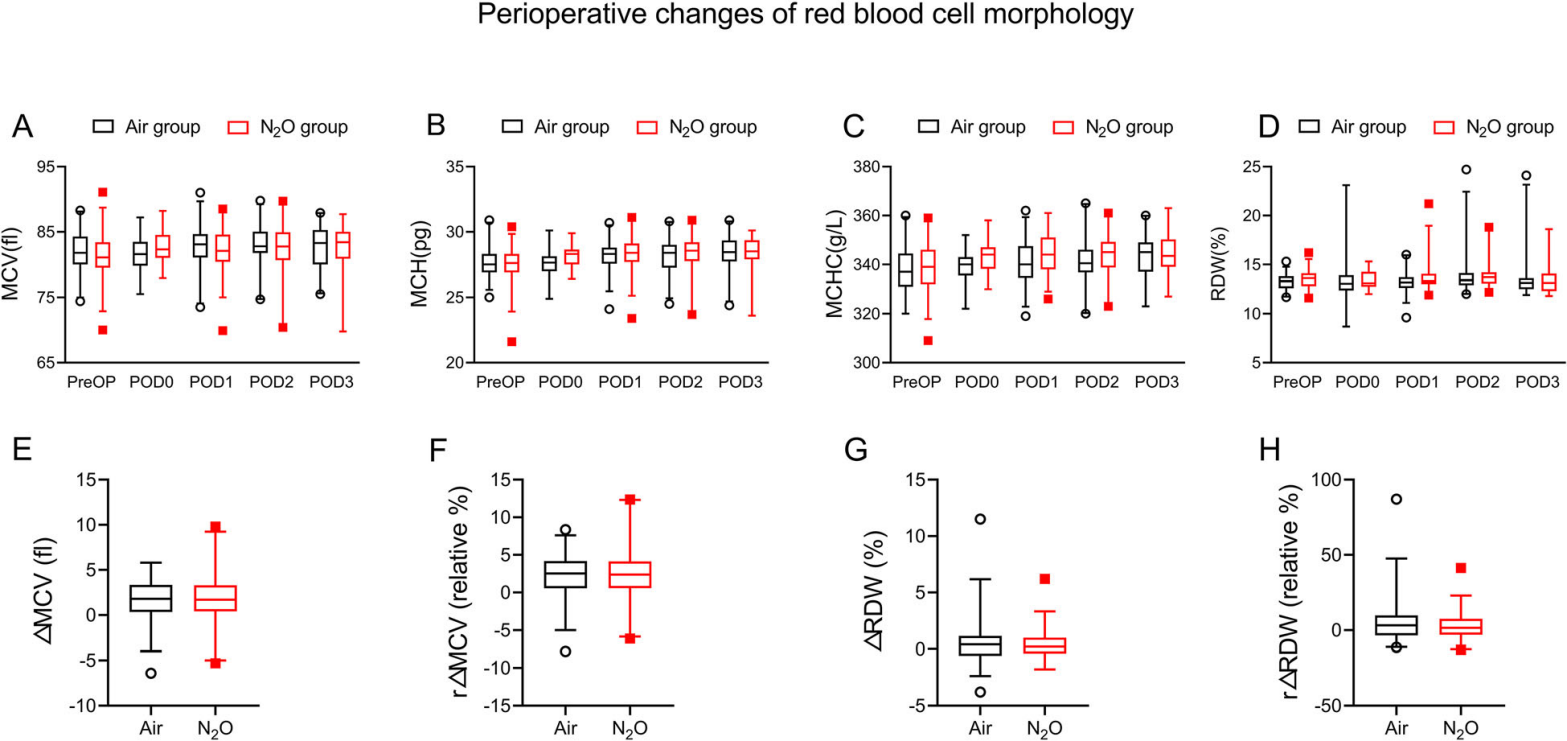
Perioperative changes of red blood cell morphology. Legend: Changes in MCV(A), MCH(B), MCHC(C) or RDW(D) were observed between the two groups. The absolute changes of MCV(E) and RDW(F) and the relative changes of MCV(G) and RDW(H) were shown separately. MCV, mean corpuscular volume; MCH, mean corpuscular hemoglobin; MCHC, mean corpuscular hemoglobin concentration; RDW, red-cell distribution width; ΔMCV, change of peak postoperative to preoperative mean corpuscular volume; ΔRDW, change of peak postoperative to preoperative red cell distribution width; rΔMCV, relative change of peak postoperative to preoperative mean corpuscular volume; rΔRDW, relative change of peak postoperative to preoperative red cell distribution width; PreOP, the last blood sample before surgery; POD, blood sample of some day after surgery

The variations of MCV, mean corpuscular hemoglobin (MCH), MCHC and RDW before and within 3 days after surgery were shown as mean and 95% confidence interval (CI) in Fig. 2(A-D). MCV peaked at 83.7(82.9–84.4) fL, and 83.2(82.4–83.9) fL and RDW peaked at 13.8% (13.4–14.2%), and 13.9% (13.6–14.2%) for the air group and the N_2_O group during the week after operation. And the absolute and relative changes within 7 days after surgery were also shown in Fig. 2(E-H). Both r∆MCV and r∆RDW were similar between the two groups. No correlation was observed between cumulative N_2_O exposure and r∆MCV (*n* = 136; r = − 0.009; 95%CI, − 0.196 to 0.173; *P* = 0.918) or r∆RDW (*n* = 136; *r* = − 0.048; 95%CI, − 0.215 to 0.115; *P* = 0.583).

Variables associated with rΔMCV based on existing literature and clinical experience were included for multiple liner regression analysis. After adjusted for age, sex, blood loss amount, intraoperative fluid infusion, allogenic transfusion amount and exclusively allogeneic transfusion percentage, N_2_O exposure was still not associated with rΔMCV (Table 4).

**Table 4:** Multiple Linear Regression Analysis for rΔMCV

Variable	B	95%CI	*P* value
Group	3.77E-4	−0.011-0.011	0.946
Age (y)	−0.006	− 0.011--0.001	0.020
Sex	0.007	−0.004-0.019	0.206
Blood loss amount (/100 mL)	0.005	8.2E-5-0.010	0.054
Intraoperative fluid infusion(/100 mL)	0.001	−0.001 − 0.004	0.332
Allogeneic transfusion amount (U)	-0.004	−0.014-0.005	0.386
Exclusively allogeneic transfusion (%)	0.011	−0.002-0.025	0.106

Group is taken to represent children with or without nitrous oxide exposure. *rΔMCV* relative changes of mean corpuscular volume

## Discussion

We conducted a comprehensive analysis of red blood cell morphology changes in pre-school age children undergoing hemivertebra resection in this retrospective study. No megaloblastic anemia or macrocytosis were observed. There was no correlation between N_2_O exposure and r∆MCV or r∆RDW.

Children with congenial scoliosis are at risk of malignant hyperthermia, so total intravenous anesthesia and ventilation with N_2_O/O_2_ or air/O_2_ are applied during surgery. We planned to do this cohort study on these young children for two reasons. On one hand, N_2_O is a wildly used inhalation anesthetic while its use remains controversial for its potential side effects related to vitamin B_12_ oxidation [12]. With impaired tetrahydrofolate and methionine synthesis, deoxyribonucleic acid (DNA) synthesis and cell cycle arrest could happen [13]. Acute megaloblastic bone marrow changes were found in seriously ill patients with N_2_O exposure for 2 to 6 h [14]. Recently several studies [2–4] have found elevated plasma homocysteine concentration under N_2_O anesthesia. Duma explored the hematological changes in 54 adolescent patients after major spinal surgery and found no association between N_2_O exposure and megaloblastic anemia. However, more studies are still needed to clarify the clinical effect like blood cell morphology or neurobehavioral changes. On the other hand, given its dose-dependent side effects [15], a study on risk population with large N_2_O exposure will help describe its clinical safety. Pre-school age children with active DNA synthesis are vulnerable and they tend to receive large amount of N_2_O anesthesia because of high respiratory rate and short drug half-time. Hemivertebrae resection are applied in patients with congenital scoliosis due to hemivertebrae and usually takes around 4 h. Current evidence has shown an exposure of N_2_O for only 2 h could increase plasma homocysteine in young children [4]. Therefore, we did this retrospective cohort study on pre-school age children undergoing hemivertebrae resection but found no significant changes in MCV and RDW after long exposure of N_2_O.

A large surgery as it is, all children suffered blood loss. Indicators in CBC such as hemoglobin, MCV and RDW are used to analyze hematological changes [16]. Hemoglobin reflects the severity of anemia, meanwhile MCV and RDW are two sensitive and specific indexes to identify the type of anemia [17]. Hemorrhagic and megaloblastic anemia are two possible conditions in our study. Hemorrhagic anemia might manifest as normocytic or microcytic anemia. N_2_O inactivates vitamin B_12_ and impairs its ability to act as a methionine synthase cofactor [18], which could lead to macrocytic changes. Meanwhile, blood loss stimulates medullary hematopoiesis, making it more vulnerable to abnormal DNA synthesis. We analyzed the peak MCV value during the 7 days after surgery and found none developed macrocytosis. However, macrocytosis might be obscured by coexisting microcytic anemia. In this situation there would be both small and large red blood cells and an increased RDW value, showing the discrete trend of red blood cell size. We analyzed RDW to show the distribution and heterogeneity of red blood cell and found no significant difference.

Blood loss and transfusion as well as fluid management may interfere hematologic changes [19], especially during large surgeries in young children [20]. Intraoperative bleeding is one of the direct causes of postoperative anemia. Although the amount of blood transfusion was greater than intraoperative blood loss in our study, there was still a high proportion of anemia after surgery. The exact volume of bleeding should be recorded by the weighed gauze and suction bottle or calculated by hemoglobin or hematocrit changes. In our study, bleeding volume was roughly recorded and actual volume was probably underestimated. Autologous or allogeneic blood transfusion might help relieve anemia while postoperative red blood cell morphology could be interfered via allogenic transfusion [10, 11]. The morphology changes of red blood cell are associated with the storge time and pre-school age children usually receive fresh red blood cell distributed by hospital blood bank. Besides, fluid management could affect hemoglobin concentrations and actual red cell loss in surgery via blood dilution or concentration. The background infusion of Ringers Lactate was set according to actual requirements during surgery and colloid or plasma could be used when necessary [21]. In this retrospective study, it was difficult to collect the exact blood loss and fluid infusion. Blood loss, the type and amount of blood transfusion and intraoperative fluid infusion were analyzed and no significant difference was found between the two groups. We included intraoperative fluid infusion amount, allogenic transfusion amount and exclusively allogenic transfusion percentage as potential confounders. After adjusting for these factors, N_2_O exposure was not associated with rΔMCV. The medium bleeding volume of 200 mL was about 15% of whole blood volume and was one of the key reasons for postoperative anemia.

Although no changes in red blood cell morphology were found, there are some limitations in our study. First, we only analyzed the morphologic changes of red blood cell for 7 days after surgery. Methionine synthase could be irreversibly damaged by N_2_O and would be de novo synthesized to recover its activity after at least 2 to 4 days depending on the protein synthesis level [22, 23]. With the damaged function of methionine synthase, abnormal DNA synthesis and hematopoieisis could happen. It takes around 188 h for proerythroblast to develop into mature erythrocyte, which can be accelerated by anemia and in young children. Duma et al. observed the hematologic effects of N_2_O for 4 days after operation and found no macrocytosis in adolescent patients [6]. In our study, many children were discharged about 4 days after surgery. We only have complete blood count results of about 1/3 children for about 1 week after surgery and found no macrocytosis. The incidence of macrocytosis could be underestimated and long-term prognosis such as infection, thrombosis, changes in the nervous and cardiovascular systems due to the elevated homocysteine need further explorations. Second, we were unable to obtain iron status, vitamin B_12_, folic acid and plasma homocysteine concentration, and *MTHFR* polymorphism in this retrospective study. N_2_O could cause more severe outcomes in patients with *MTHFR C677T* or *A1298C* gene variant [24]. We could better describe the effect of N_2_O with those above. Third, the accurate bleeding volume was unable to obtain and blood loss was probably underestimated. A 15% loss of whole blood volume in such young children was one of the main reasons of postoperative anemia and this might obscure the side effect of N_2_O exposure. A prospective randomized controlled trial or cohort study with accurate recording of blood loss and fluid infusion amount and pre- and post-operative iron, vitamin B_12_, folic acid and homocysteine concentration might help to better clarify the effect of N_2_O anesthesia on red blood cell morphology changes and long-term prognosis such as neurobehavioral and cardiovascular changes.

## Conclusions

In conclusion, our results suggest that pre-school age children undergoing hemivertebra resection could receive N_2_O anesthesia for 4 h with a low probability of megaloblastic anemia or other changes in red blood cell morphology. More studies should be done to clarify the long-term outcomes under N_2_O anesthesia in pediatric patients.

## Data Availability

All main data were presented within the manuscript and the datasets used an analyzed during the current study are available from the corresponding author on reasonable request.

## Abbreviations

N_2_O: Nitrous oxide
DNA: Deoxyribonucleic acid
CBC: Complete blood count
MCV: Mean corpuscular volume
RDW: Red cell distribution width
r∆MCV: Relative changes of MCV
r∆RDW: Relative changes of RDW
MCHC: Mean corpuscular hemoglobin concentration
CI: Confidence interval
MCH: Mean corpuscular hemoglobin
